# Responses of yield, quality and water use efficiency of potato grown under different drip irrigation and nitrogen levels

**DOI:** 10.1038/s41598-023-36934-3

**Published:** 2023-06-19

**Authors:** Mustafa Akkamis, Sevgi Caliskan

**Affiliations:** Department of Plant Production and Technologies, Faculty of Agricultural Sciences and Technologies, Nigde Omer Halisdemir University, Nigde, Turkey

**Keywords:** Physiology, Plant sciences

## Abstract

Proper irrigation and fertilization are essential for achieve high tuber yield and quality in potato production. However, the high cost of these inputs necessitate optimization of their use to improve both water use efficiency and crop productivity. This study aimed to investigate the impact of irrigation and nitrogen fertilization on potato yield, quality and water use efficiency. The research included different drip irrigation treatments (100%, 66%, and 33% of field capacity) and nitrogen levels: 0 (N0), 100 (N1), 200 (N2), 300 (N3), 400 (N4) and 500 (N5) kg N ha^−1^. The results indicated that potato yield and growth were more sensitive to irrigation treatment than nitrogen levels. Full irrigation with 300 kg N ha^−1^ produced the highest total tuber yield, while low irrigation treatments resulted in significantly lower yields. In contrast, the 66% field capacity irrigation treatment consistently had the highest water use efficiency in both years of the study. Furthermore, the study showed that the quality characteristics of the tubers were negatively impacted by full irrigation treatments compared to low irrigation. These findings suggest that with appropriate irrigation and nitrogen application, potatoes can be produced with acceptable yields while conserving water and minimizing nitrogen use. This research emphasizes the importance of optimizing inputs to improve water use efficiency and yield productivity while reducing water. As a result, obtaining useful information on crop management for farmers to make informed decisions may be possible by achieving optimal irrigation and nitrogen levels.

## Introduction

Irrigation and nitrogen management (N) are important factors affecting potato (*Solanum tuberosum* L.) yield, quality, and net profit^1^. Potato yields are maximized when the soil moisture is consistently maintained at an optimal level and adequate nitrogen supply is provided^2^. A sufficient nitrogen is necessary for the high growth rate of potato plants, leading to increased tuber yield but decreased specific gravity. Insufficient nitrogen results in reduced leaf area and tuber size due to early leaf drop, while excessive nitrogen content in the soil leads to an increase in plant dry matter content and a decrease in the duration of tuber growth^3–8^.

Applying nitrogen at the right rate, time and place increases N efficiency. Potato need nitrogen most during the tuber growth period. Approximately 58–70% of N during the entire production period is taken at this stage of development^9^. When nitrogen is applied to the plant in the most appropriate form and amount, it has a positive effect on growth and plant development. However, excessive use of nitrogen negatively affects the resistance of the plant against diseases and pests. Due to low nitrogen in the tuber formation stage, drying of the tuber and old leaves occurs and therefore reduces tuber development.

The limited root system of potato requires the application of nitrogen fertilizers since the plant has a low utilization capacity for nitrogen. Therefore, effective management of irrigation and nitrogen fertilization is crucial for optimal growth and development of the crop, with due consideration for attaining maximum yield and quality of the harvest^10^. The water consumption of potato ranges from 500 to 700 mm depending on the climatic factors. To achieve high yields in potato, being an exceptionally moisture-sensitive crop, must maintain an available water content of not less than 65%^11,12^. During the period from the initiation of tuber formation to 15 days prior to harvest, the potato displays its greatest demand for water. In the absence of proper irrigation during this stage, the tubers may exhibit secondary growth. While irrigation promotes an increase in average tuber weight, it may not necessarily lead to a higher number of tubers per plant^13^.

The irrigation method utilized in potato cultivation varies based on the region and availability of water resources. In the potato-growing regions of Türkiye, sprinkler irrigation is the predominant method, although drip irrigation methods have also gained popularity^14^. Nitrogen can infiltrate under the root through irrigation and precipitation. Accordingly, fertilizers and chemicals that cannot be taken by the plant move underground with the water. Precipitation and irrigation are instrumental in determining the movement rate of such chemical through to the soil surface, which can be used to manage their submergence Therefore, controlled irrigation management is vital for regulation the transport of chemicals and nutrients. Proper application of irrigation method can also facilitate nitrogen uptake, thus minimizing potential seepage losses below the root zone^15^. Although the information in the literature on irrigation and nitrogen management is conflicting, tuber yield and quality are affected by N and irrigation applications. Proper management of nitrogen and water is necessary to achieve growth and marketable tuber. Incomplete irrigation creates differential effects on nutrient uptake, growth, and yield. Nitrogen can replace deficient water, and effective nitrogen management can mitigate yield loss due to the under-irrigation.

Two potential strategies to enhance water utilization efficiency in potato production are the implementation of appropriate irrigation scheduling and the utilization of drip irrigation. Effective nitrogen management can also significantly contribute to improved plant growth and yield^16^. Therefore, attention must be paid to N and water management for the potato to provide quality and marketable tubers. In many parts of the world, various studies were conducted on irrigation and fertilization of potato. The most limiting nutrient for potato growth, the need for nitrogen varies greatly with climate, soil, variety, irrigation, and cultural practices. Accordingly, the present study aims to investigate the effects of different irrigation and nitrogen fertilization levels on yield and quality of potato plants cultivated under drip irrigation conditions.

## Materials and methods

### Site description

Field trials were conducted during the years 2021 and 2022 at research area (N37° 94’, E34° 96’) Faculty of Agricultural Sciences and Technologies, Nigde Ömer Halisdemir University, Türkiye. The experimental site is at an altitude of 1299 m above sea level and receives an average of 343 mm of precipitation annually. In both years, a rainfall recorder (Turkish State Meteorological Service Nigde Meteorology Station) was used to measure the precipitation during the growing season. At the sowing time, the soil bulk density of experimental field (0–40 cm) was 1.11 g cm^−3^, field water capacity was 31%, soil pH value was 7.95, and the soil available nitrogen, phosphorus and potassium contents were 0.138%, 10.85 mg kg^−1^, and 201.19 mg kg^−1^, respectively.

### Experimental design

In each year, the field experiments were conducted according to the split-plot design with 18 sub-plots replicated four times. The treatments were comprised of three irrigation water levels (100% = S100, 66% = S66, 33% = S33 field capacity) and six nitrogen levels (0 = N0, 100 = N1, 200 = N2, 300 = N3, 400 = N4, 500 = N5 kg ha^−1^). The nitrogen levels were randomized in main plots whereas the irrigation levels were randomized in sub-plots. ‘Agria’ was used as a variety in experiments during both the years. The disease-free seed tubers of this variety were obtained from Doga Seed Company, Nevsehir, Türkiye. The seeds were sown on the ridge tops with a sowing machine on May 13, 2021, and May 29, 2022, and were harvested on October 4, 2021, and on September 27, 2022 during the first and second year of study, respectively. The entire field (65.1 m × 29.4 m) was divided into four blocks (replications) and each block measured 65.1 m × 5.1 m. Among the blocks, an area measuring 3 m was kept unplanted to facilitate data recording and to prevent irrigation applications from affecting each other. Each block was divided into six main plots. The main plots were consisted of 12 rows and sub-plots consisted of four rows. Two rows between the main plots and one row between the sub-plots were kept unplanted. Experimental research on plants, field studies, collection of plant material and irrigation practices were carried out in accordance with the Standards of the Ministry of Agriculture of the Republic of Türkiye.

#### Irrigation management and crop water consumption

After planting potato seeds, drip irrigation systems were placed on the field. Immediately after planting, emitters at 30 cm intervals were placed in a drip tape with an emitter flow rate of 4 L h^-1^. A system consisting of a screen filter, fertilizer tank, a valve and two pressure gauges was used to measure the irrigation amount and control the pressure. Irrigation started on May 13, 2021 and May 29, 2022. To measure the field capacity, soil samples were taken from 0–20 to 20–40 cm depths with a soil digger. Field capacity was measured as the amount of water retained in a saturated soil after 2–3 days of gravity drainage. Volumetric moisture content was calculated gravimetrically. The amount of irrigation water applied to each plot was calculated with the following Eq. (1)^17^.

1 $$\mathrm{Irrigation Requirement}=[\frac{(\mathrm{Fc}-\mathrm{Sm})}{100}\times {\mathrm{R}}_{\mathrm{d}}]\times \mathrm{Pa}\times \mathrm{Pw}$$

where “Fc” is field capacity (31%), ‘’Sm’’ is soil moisture before irrigation (%), “R_d_” is root depth (mm), “Pa” is plot area (m^2^) and “Pw” is wetted soil percentage.

Water was applied when the soil moisture decreased by 30–40% of the field capacity, separate irrigation was applied to each nitrogen plot. To monitor the soil moisture content (%), soil samples were taken every 3–4 days from the full irrigation plot (100% Fc) of each nitrogen application, and the gravimetric method (g/g) soil moisture measurement was performed.

Crop water consumption (ETc, mm) was calculated at 15-day intervals for each nitrogen level using the soil water balance (Eq. 2)^18^.

2 $$\mathrm{ETc}=\mathrm{I}+\mathrm{P }\pm \Delta \mathrm{S}-\mathrm{D}-\mathrm{R}$$

where I is the irrigation water (mm), P is the rainfall (mm), ∆S is the change in soil water storage (mm 60 cm^−1^) and D is the deep percolation (mm), R is the runoff (mm). There was no Runoff as adequate weirs were provided. Deep percolation was accepted zero when soil moisture was less than field capacity. When soil moisture after irrigation or precipitation surpassed field capacity, deep percolation was evaluated as the difference between field capacity and soil moisture plus irrigation/precipitation^19^.

#### Water use efficiency (WUE)

WUE (kg mm^−1^ ha^−1^) were calculated using Eqs. (3) described by Hou et al.^20^

3 $$\mathrm{WUE}=\frac{\mathrm{Y}}{\mathrm{Et}}$$

where Y is the crop yield, ET is the evapotranspiration during the entire growth period.

#### Fertilization management

After the completion of the land preparation, fertilizers were applied. P_2_0_5_—125 kg ha^−1^ K_2_0—150 kg ha^−1^ were surface spread prior to planting. Likewise, half of the nitrogen dose was applied during planting and the remaining half was applied during tuber bulking. Planting was performed with a distance of 30 cm between plants and 70 cm between rows. Plant protection practices were carried out throughout the entire growing season. Potato seeds separated as seeds were sprayed before planting, with Thiamethoxam active ingredient, against pests after emergence. At the growth stage, fungicide against blight disease were also used as per requirements.

### Data collection

#### Yield and growth parameters

The growth parameters of plants in each replicated plot, including the number of tuber plant^−1^, number of stems^−1^, and height of plants (cm) were noted. Tubers in each plot were first classified, then counted and finally weighed. Classifications: Diameter greater than or equal to 45 mm—class 1; greater than 25 and less than 45 cm—class 2; Less than or equal to 25 mm—class 3. 1st, 2nd, and 3rd class yields of tubers were added and ton ha^−1^ weights were calculated.

#### Tuber quality parameters

At harvest, tuber dry matter (TDM) and specific gravity (SG) were measured each year on all treatments. TDM and SG of treatments were measured by Martin Lishmans’s digital potato hydrometer. TDM and SG were measured with approximately 2.5 kg of clean, raw tubers from each treatment. Starch concentration was calculated using the underwater weight of the tubers with Eq. (4)^21^.

4 $$\mathrm{Starch }(\mathrm{\% FW}) = -183 + (184\mathrm{ x SG})$$

#### Statistical analysis

All data were subjected to experimental design analysis of variance (ANOVA) to evaluate the effects of treatments on yield, growth components and tuber quality of potato. The SAS Institute (Version 9, Cary, NC, USA, 2002) was used to perform the analysis of variance. Comparison of the means was obtained using the least significant difference (LSD) at the 5% probability level.

## Results and discussion

### Meteorological parameters

**Figure 1:**
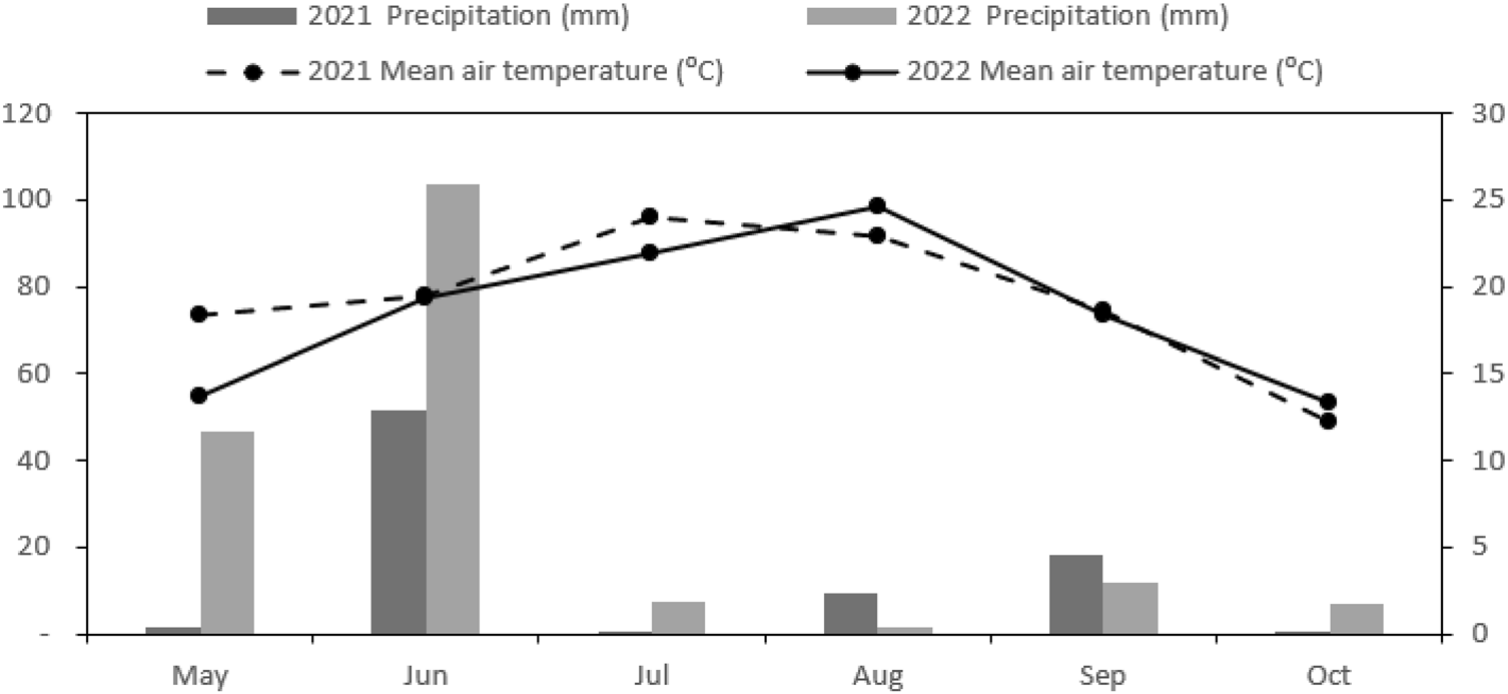
Mean air temperature and precipitation during the growing season of 2021–2022.

The mean monthly meteorological parameters for both years are presented in Fig. 1. The maximum mean monthly temperature was observed during the tuber formation in 2021 and tuber expansion month in 2022. Precipitation levels during the 2021 growing season were lower compared to the subsequent season (2022), but not unevenly distributed, with majority of the rain occuring in June (Fig. 1). The total precipitation during the potato growing seasons in 2021 and 2022 was 82.20 mm and 177.60 mm, respectively (Fig. 1). Morever, lower temperature values prior to planting in 2022 resulted in a delay in the planting time.

### Irrigation water applied and crop water consumption

**Figure 2:**
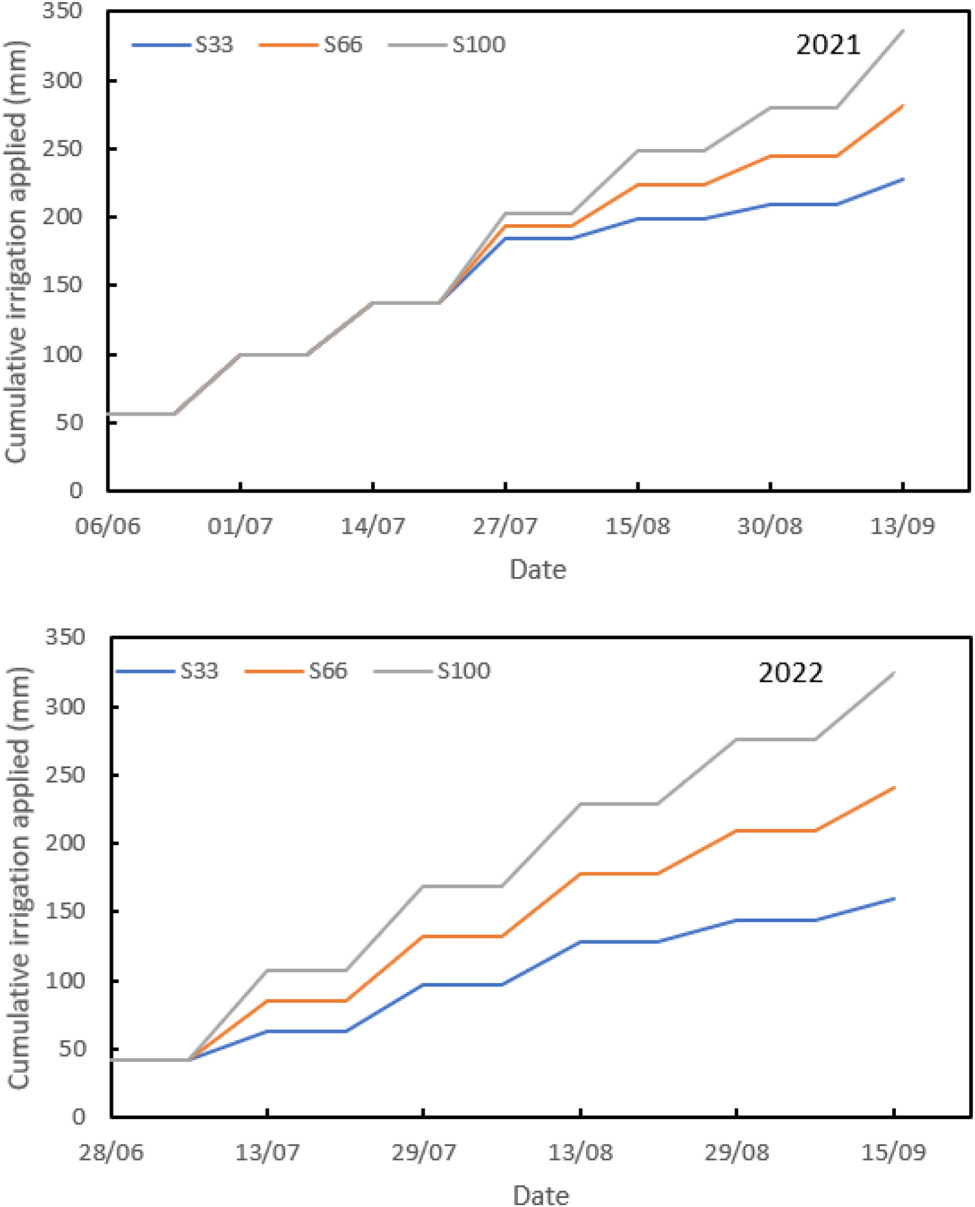
Water applied amount for each irrigation level during the years 2021 and 2022.

The total water application for each treatment and growing season is shown in Fig. 2. The average total irrigation applied to the crop was 227.70, 280.90, and 335.70 mm for S33, S66, and S100 treatments in 2021, respectively and 160.10, 241.00, and 324.3 mm for S33, S66, and S100 treatments in 2022, respectively. The first-year irrigation amount was higher than the second year because the water deficit period was delayed in the first year. The seasonal crop water consumption (ETc) values determined are given in Table 1. Seasonal average ET_c_ values varied between 181.78 and 289.79 mm in 2021 and between 234.48 and 369.14 mm in 2022.

**Table 1:** Crop water consumption (ETc) for each treatment (mm) in both year.

Years	Treatments	N0	N1	N2	N3	N4	N5	Average
2021	S33	144.87	202.72	202.10	194.05	182.48	164.47	181.78
	S66	187.59	249.20	255.94	258.00	242.25	216.91	234.98
	S100	231.60	297.09	311.40	323.89	303.84	270.93	289.79
2022	S33	227.67	246.55	235.75	219.29	250.24	227.40	234.48
	S66	268.81	289.91	311.52	283.31	314.23	292.58	293.39
	S100	344.75	401.58	386.97	348.60	395.19	337.78	369.14

N0 = 0 kg ha^−1^; N1 = 100 kg ha^−1^; N2 = 200 kg ha^−1^; N3 = 300 kg ha^−1^; N4 = 400 kg ha^−1^; N5 = 500 kg ha^−1^; S33 = 33% FC; S66 = 66% FC; S100 = 100% FC.

### Yield and growth parameters

The potato tuber yields exhibited significant variation in response to different levels of nitrogen and drip irrigation, as indicated in Table 2. The S100 irrigation level achieved highest tuber yield under all N levels. Notably average yield was more sensitive at irrigation levels than nitrogen levels. Gradual water deficit resulted in an average reduction in total yield of 14.9% in 2021 and 10.5% in 2022 with a reduction in irrigation water at the S66 level, whereas the application of S33, which represented a lower amount of water, led to a 37.2% decrease in potato yield in 2021 and 39.8% in 2022. The findings of Badr et al.^2^ support this observation, as full irrigation resulted in the highest tuber yield under all nitrogen levels. Moreover, as the amount of irrigation water decreased, the total yield reduced by an average of 7.8% with a 20% reduction in irrigation water. Meanwhile, a decrease in potato yield by 27.3% and 44.6% was observed when 40% and 60% less water was applied, respectively. It was further noted that while the total yield increased up to the N3 level with an increase in the amount of nitrogen, it decreased beyond this point. The decrease in tuber yield beyond a certain level of nitrogen was attributed to the plant experiencing stress, ultimately leading to a decrease in yield.The application of irrigation and nitrogen fertilizer rates individually have demonstrated significant impacts on the growth attributes of potato crops, with a notable interaction effect between the two factors. It was observed that the maximum growth attributes of potato crops were achieved when irrigated with S100, while the minimum values of plant height were recorded in plots irrigated with S33, across both years. Similarly, an increase in nitrogen application rates was positively correlated with plant height. In particular, the highest plant height was recorded in response to N4 treatment in 2021 and N1 treatment in 2022 (Table 2). Notably, the response of plants to water scarcity in nitrogen fertilization presents a crucial factor in understanding how plants allocate their resources to aboveground and underground organs, thereby influencing their growth and development. Therefore, the association with plant height was observed to be slightly but consistently shorter in N0 plants. This observation aligns with findings reported by Wang et al.^22^ where N-fertilization treatments were found to result in significantly higher plant heights in full irrigation treatments than in other irrigation treatments. Moreover, Kumar et al.^23^ have similarly noted that plant height tends to increase with increasing N doses up to 180 kg N ha^−1^. Furthermore, Yuan et al.^13^ indicated that plant height was observed to increase proportionately with the increasing amount of irrigation from Ep0.25 to Ep1.25.

**Table 2:** Total yield and growth parameters of potato different irrigation levels and nitrogen fertilization.

Treatments	Tuber per plant		Stem per plant		Plant height (cm)		Tuber yield (t ha^−1^)	
Nitrogen fertilization	2021	2022	2021	2022	2021	2022	2021	2022
N0	2.57a	5.74c	2.98bc	5.80a	62.08c	60.56a	19.17a	21.36a
N1	3.16a	6.77ab	2.80c	5.41ab	64.36bc	63.15a	22.77a	21.95a
N2	2.85a	6.43bc	3.08bc	5.16bc	63.65bc	60.73a	24.68a	22.17a
N3	3.17a	7.18a	3.15ab	4.80cd	64.18bc	54.78a	26.37a	22.36a
N4	3.19a	6.01c	3.43a	4.50d	67.73a	58.05a	25.14a	20.33a
N5	3.60a	6.10bc	3.23ab	4.85cd	65.18ab	55.66a	24.43a	18.92a
*Irrigation levels*								
S33	2.91a	5.85b	2.75b	5.12ab	61.44c	50.88c	18.06c	16.15c
S66	3.07a	6.76a	3.22a	5.36a	65.05b	60.85b	24.46b	24.01b
S100	3.29a	6.51a	3.38a	4.77b	67.10a	64.72a	28.76a	26.84a
*Significance*								
N	ns	**	*	**	**	ns	ns	ns
I	ns	**	**	*	**	**	**	**
N × I	ns	ns	ns	ns	*	ns	ns	ns

**p < 0.01, *p < 0.05. ns = non-significant. N: Nitrogen, I: Irrigation. Mean values within the same columns by different letters are significantly different. N0 = 0 kg ha^−1^; N1 = 100 kg ha^−1^; N2 = 200 kg ha^−1^; N3 = 300 kg ha^−1^; N4 = 400 kg ha^−1^; N5 = 500 kg ha^−1^; S33 = 33% FC; S66 = 66% FC; S100 = 100% FC.

According to the results presented in Table 2, there were no significant differences in the number of tubers per plant for nitrogen fertilization and irrigation in 2021. However, in 2022, there were significant differences observed. (Table 2). The number of tubers tended to increase with an increase in nitrogen content. Nevertheless, decreased under water-deficit conditions. In particular, the irrigation level of S100 in 2021 resulted in a higher number of tubers per plant, while the irrigation level of S66 in 2022 resulted in the highest number of tubers per plant. On the other hand, the number of tubers decreased under N0 in both years, with the highest tuber number obtained from the N5 treatment in 2021 and the N3 treatment in 2022. Onder et al.^11^ reported that irrigation levels of 66% of full irrigation resulted in the highest number of tubers per plant. Mattar et al.^24^ observed that the number of tubers per plant was highest with full irrigation. However, in contrast, Ghasemi et al.^25^ and Fandika et al.^26^ indicated that the effect of irrigation water on the number of tubers was not significant. Previous studies have found that water stress reduces the number of tubers per plant. Also, the lowest tuber number per plant was found in 0 kg ha^−1^ N application, and other treatments were in the same statistical group by Güler^27^. Similarly, Ahmed et al.^28^ reported a reduced number of tubers per plant, and the lowest number was obtained when using low application rates of 130 and 180 kg N/fed.

The number of stems per plant varied significantly in response to different N fertilization and irrigation levels. When N applications were evaluated, the highest stem values per plant were obtained from N4 application in 2021 and from N0 application in 2022 (Table 2). In addition, the increase in the amount of nitrogen caused an increase in the number of stems in 2021 and a decrease in 2022. The reason is that stem number is not affected much by mineral nutrients. Stem numbers per plant were affected significantly by varied irrigation levels. The irrigation level of S100 resulted in a higher stem number per plant in 2021, while the irrigation level of S66 resulted in the highest number of stems per plant in 2022. Contrary to our research, Adhikari and Rana^29^ and Kumar et al.^23^ showed that the effect of various irrigation levels on the number of stems per hill was not significant. Factors such as the storage conditions of tubers, the number of viable sprouts during planting, sprout damage and growing conditions during planting, physiological age of the seed tuber and tuber size also affect the number of stems^30^.

### Tuber quality parameters

The effects of nitrogen and irrigation on tuber dry matter (TDM), specific gravity (SG) and starch in both years are shown in Table 3. The mean values determined a significant difference (*P* < 0.01) of tuber TDM, SG and starch in irrigation and nitrogen levels in both years. Regarding the two-year analysis, the highest dry matter content was achieved with N2 treatment for nitrogen. The findings suggest that the amount of dry matter in irrigation levels tends to decrease with increasing irrigation. TDM was higher with deficit irrigation than with full irrigation in 2022. However, there was no change in 2021. Several research reports have observed a reduction in tuber dry matter with increasing N application rates^30,31^. Others have demonstrated that increasing N fertilization had no significant effect on tuber dry matter^32^. In the current study, an increasing trend in TDM was observed with increasing N treatments in 2021, while no significant changes were observed in 2022. It is possible that the higher amount of irrigation in the first year compared to the second year led to excessive nitrogen intake, which in turn contributed to the increase in TDM.

**Table 3:** Effect of different nitrogen and irrigation levels on tuber quality parameters.

Treatments	Dry matter (%)		Specific gravity (g cm^−3^)		Starch (%)	
Nitrogen fertilization	2021	2022	2021	2022	2021	2022
N0	18.86c	19.63a	1.072e	1.077a	13.23e	14.13a
N1	18.90c	19.46a	1.074d	1.076a	13.60d	13.99a
N2	20.72a	19.31a	1.083a	1.075a	15.25a	13.87a
N3	19.60b	19.09a	1.077c	1.074a	14.09c	13.67a
N4	19.31b	19.35a	1.076cd	1.075a	13.90cd	13.81a
N5	20.39a	19.43a	1.080b	1.076a	14.76b	13.95a
*Irrigation levels*						
S33	19.66a	19.77a	1.077a	1.077a	14.13a	14.25a
S66	19.59a	19.57a	1.077a	1.077a	14.12a	14.10a
S100	19.64a	18.79b	1.077a	1.073b	14.15a	13.36b
*Significance*						
N	**	ns	**	ns	**	ns
I	ns	**	ns	**	ns	**
(NXI)	ns	ns	ns	ns	ns	ns

**p < 0.01, *p < 0.05. ns = non-significant. N: Nitrogen, I: Irrigation. Mean values within the same columns by different letters are significantly different. N0 = 0 kg ha^−1^; N1 = 100 kg ha^−1^; N2 = 200 kg ha^−1^; N3 = 300 kg ha^−1^; N4 = 400 kg ha^−1^; N5 = 500 kg ha^−1^; S33 = 33% FC; S66 = 66% FC; S100 = 100% FC.

SG showed a tendency to decrease with increasing applied in 2022, with less irrigation water produced higher SG tubers. However, in 2021 SG was not affected by rising water levels. Over the two years, the highest SG was achieved with S100 for irrigation and N2 for nitrogen levels (Table 3). SG is an important quality factors for processing potato. There is a range of specific gravities that is considered optimal. Many factors such as climatic conditions and N fertilization affect tuber SG^2^. Rising of SG with increasing N application might be attributed to the increase in dry matter content, as there is high correlation between SG in tubers and dry matter. Furthermore, deficit irrigation after tuber initiation in the middle of the growing season creates tubers with reduced SG. Yuan et al.^13^ reported that as applied water increased, SG tended to decrease. In addition. Alva et al.^1^ explained that nitrogen increases SG but is not affected by irrigation.

The results revealed that increasing N application rate led to an increase in starch content, while no significant difference was observed in the irrigation regimes in 2021. However, in 2022, there was no significant difference in starch content between different nitrogen levels. It was also noted that starch accumulation was positively correlated with the amount of irrigation applied, and the highest starch content was observed in the S100 treatment for irrigation and N2 treatment for nitrogen levels. (Table 3). These results agree with previous studies indicating a positive correlation between water content and starch accumulation in potato tubers. Specifically, lower water availability has been associated with increased starch content, potentially due to reduced cell size resulting from water stress^22,33^. However, there are some contrasting reports suggesting that increased nitrogen fertilizer rates could lead to a reduction in starch content. Considering these findings, proper management of irrigation and nitrogen application is essential for maximizing starch accumulation in potato tubers^34^.

### Water use efficiency

**Figure 3:**
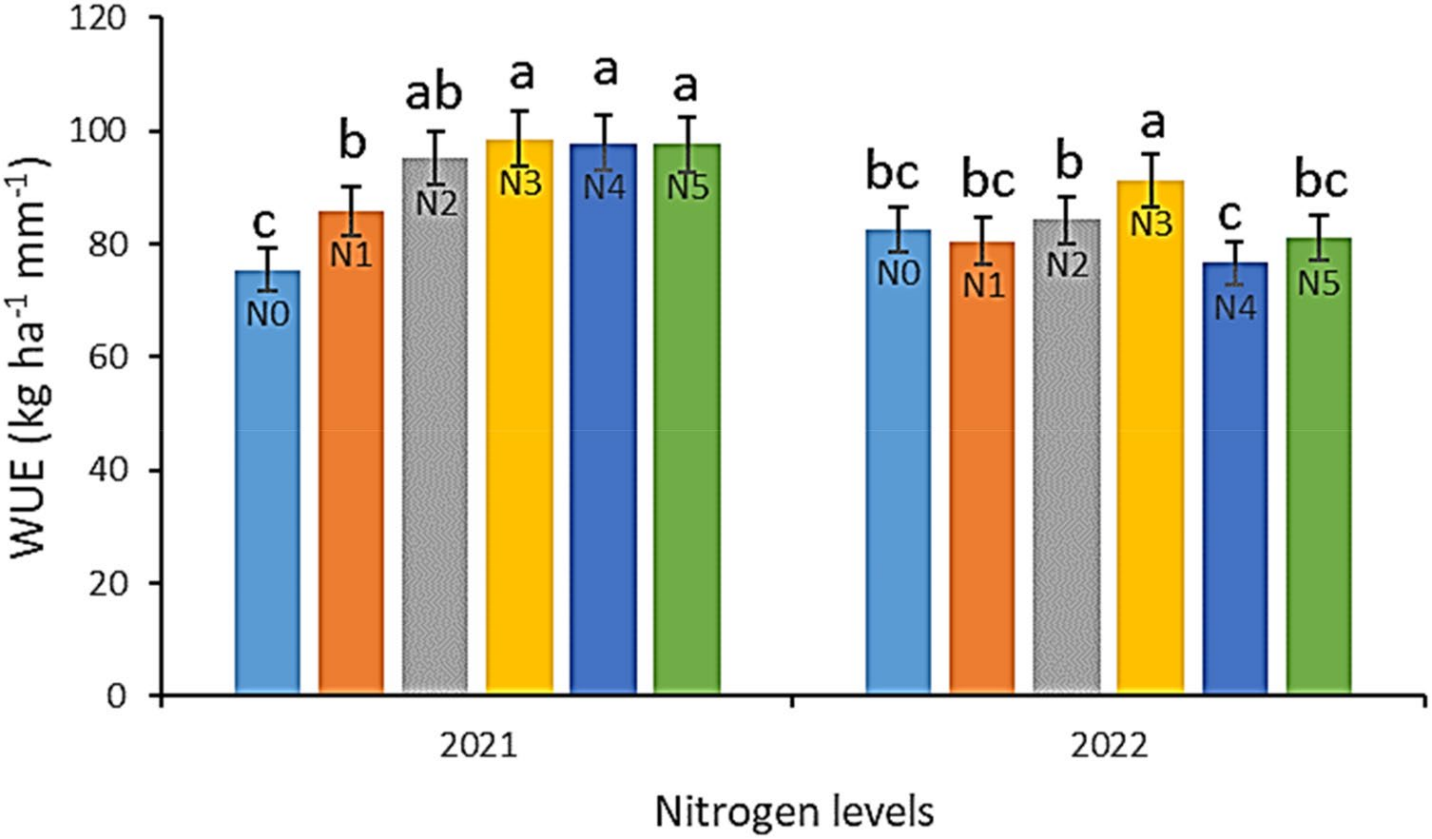
Effect of different nitrogen levels on water use efficiency of potato. Different letters are significantly different. (N0 = 0 kg ha^−1^; N1 = 100 kg ha^−1^; N2 = 200 kg ha^−1^; N3 = 300 kg ha^−1^; N4 = 400 kg ha^−1^; N5 = 500 kg ha^−1^).

**Figure 4:**
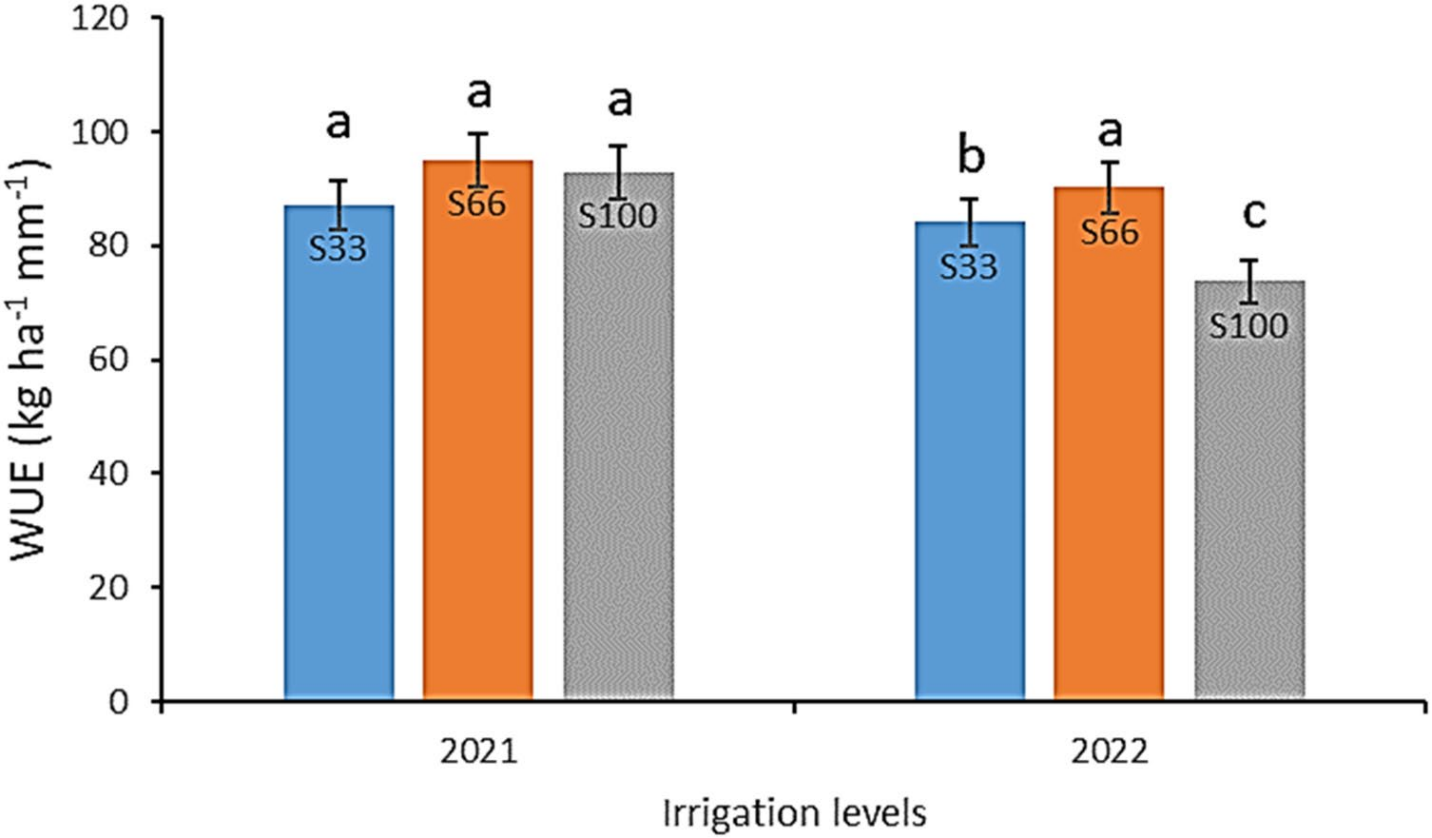
Effect of irrigation levels on water use efficiency of potato. Different letters are significantly different (S33 = 33% FC; S66 = 66% FC; S100 = 100% FC).

The water use efficiency (WUE) of potato crops was affected by different irrigation and nitrogen levels during the growing season in each year as shown in Figs. 3 and 4. There was a significant difference between the years. Comparisons among mean values of the water levels treatments indicated that S66 had the highest mean value of WUE in both years, followed by S33, S100 and S100, S33 in 2021 and 2022, respectively. The effect of irrigation levels on WUE may depend on the level of water stress at different growth periods. In low water stress conditions, transpiration decreases more than photosynthesis under the condition of slight closure of stomata, and as a result WUE increases^35^.

The nitrogen levels had a significant effect on WUE, with potato crops exhibiting an increase in WUE up to the N3 level, followed by a decrease in WUE after this threshold in both years (Fig. 3). Moreover, the findings suggest that WUE was positively correlated with irrigation levels, whereby an increase in water levels resulted in a concomitant increase in WUE. Under water-deficient conditions, WUE was found to be enhanced, which is in line with previous reports that have documented an increase in WUE relative to an increase in water stress^2,36,37^.

### *Relationship between tuber yield, irrigation levels and *Etc

**Figure 5:**
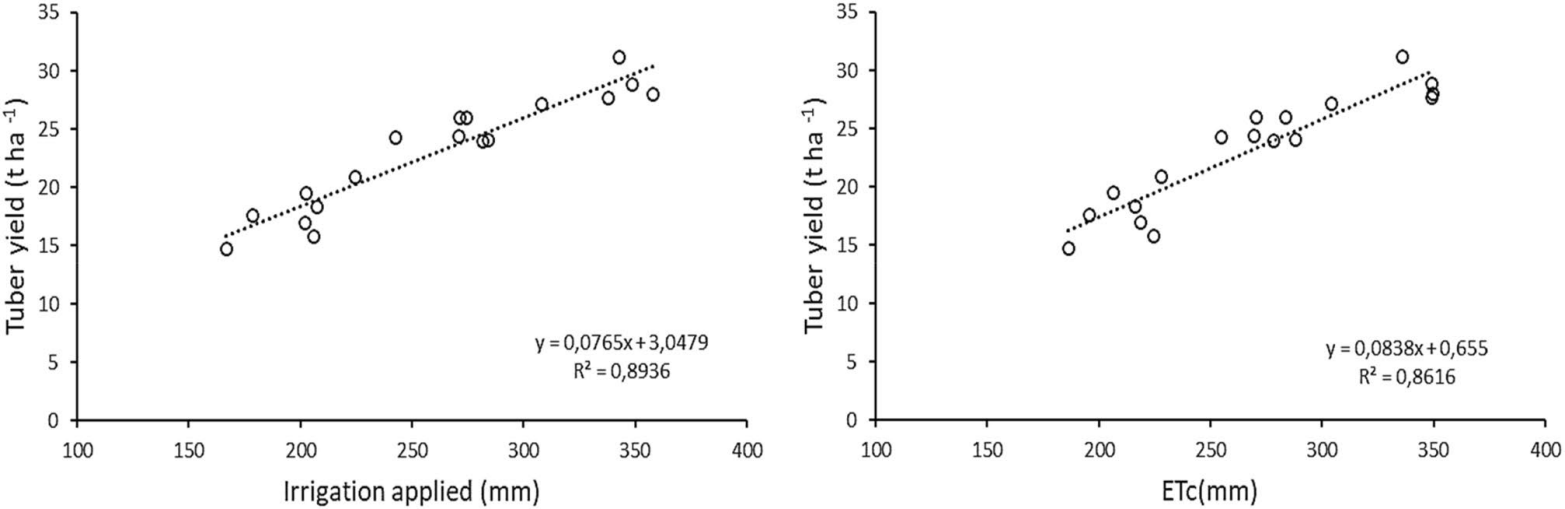
Relationship between tuber yield, irrigation applied and Etc of potato.

Linear regression analysis was utilized to determine the total amount of irrigation applied in tons per hectare. The relationship between potato tuber yield and applied water is presented in Fig. 5, showing an increase in yield with increasing irrigation application. The linear regressions between irrigation applied and tuber yield were found to be significant. Moreover, significant linear relationships were also observed between potato tuber yield and ETc, as depicted in Fig. 5. Previous research has indicated that potato yield responds linearly to the quantity of water applied^2,11,38^. Ünlü et al.^39^ reported that depending on the irrigation regimes, evaporation and tuber yield were positively affected by nitrogen fertilizer. Badr et al.^2^ indicated that the relationships between tuber yield and crop ET were linear. The relationship between potato yield and ET serves to elucidate the strength of the yield's linear increase with ET^40,41^. The aim of irrigation applications is to achieve maximum efficiency through optimal irrigation application and appropriate irrigation regimes^42^.

## Conclusions

Effective management of irrigation and nitrogen is crucial for optimizing potato yield, quality, and water use efficiency. This study highlights that full irrigation with an application rate of 300 kg N/ha resulted in the highest tuber yield, indicating the importance of providing adequate water and nitrogen for optimal crop performance. However, it is important to note that excessive nitrogen levels can have detrimental effects on potato yield and quality. Therefore, careful attention to irrigation and nitrogen levels is necessary to achieve the desired outcomes. The findings of this study provide valuable insights for potato growers and agricultural practitioners. By implementing appropriate irrigation and nitrogen management strategies, farmers can maximize productivity and quality while minimizing the use of water resources. This is particularly relevant in regions where water scarcity and environmental concerns are significant challenges. Furthermore, this research emphasizes the need for sustainable crop management practices. Balancing the application of water and nitrogen is essential not only for achieving optimal yields but also for conserving water resources and minimizing nutrient losses. By adopting precise irrigation scheduling and optimizing nitrogen application rates, farmers can enhance water use efficiency and reduce potential negative environmental impacts. To further advance our understanding, future research should focus on assessing the long-term effects of different irrigation and nitrogen management strategies on potato crops. This would enable the development of more comprehensive guidelines and recommendations for growers to make informed decisions regarding irrigation and nitrogen application.

## Data Availability

The datasets generated and/or analysed during the current study are not publicly available due as it is part of the corresponding author’s doctoral thesis, and the other part of the study is in progress but are available from the corresponding author on reasonable request.
